# Supplementary material for: Cardiorespiratory Fitness Mediates Cognitive Performance in Chronic Heart Failure Patients and Heart Transplant Recipients
Source: Int J Environ Res Public Health. 2020 Nov 19;17(22):8591. doi: 10.3390/ijerph17228591 (PMC7699401; doi:10.3390/ijerph17228591)
Supplement: Supplementary file 1 [file ijerph-17-08591-s001.zip › ijerph-970955-tables.docx]

| **Supplementary Materials**  **Table S1**: Exclusion and inclusion criteria for HC, HT recipients and HF patients | | |
| --- | --- | --- |
|  | Inclusion criteria | Exclusion criteria |
| HC | - Age >18 years - No evidence of coronary heart disease - Able to perform a cardiopulmonary exercise test | - Significant resting ECG abnormality - History of ventricular arrhythmias or congestive heart failure - Stroke or uncontrolled hypertension - Recent modification of medication (<2 weeks) - Musculoskeletal conditions making exercise on ergocycle difficult or contraindicated - Neurological diseases and/or MMSE score below 26 |
| HT recipients | - Age >18 years - Heart transplantation for more than 1 year - Stable clinical condition including optimal medication and with no cardiac allograft rejection - Able to perform a cardiopulmonary exercise test |  |
| HF patients | - Age >18 years - LVEF <40% (measured within 6 months of their enrolment by MUGA Scan, echo or radiological ventriculography) - NYHA functional class I-III - Optimal therapy at stable doses including a beta-blocker and an ACE inhibitor or ARA for at least 6 weeks prior to investigation - Able to perform a symptom-limited maximal exercise test, capacity to sign the informed consent form | - Recent cardiovascular event (< 3 months) - Any relative or absolute contraindications to exercise among patients with stable chronic HF according to current recommendations - Chronic atrial fibrillation - HF secondary to significant uncorrected primary valvular disease (except for mitral regurgitation secondary to LV dysfunction) - HF secondary to congenital heart disease or obstructive cardiomyopathy - Severe non-revascularizable coronary disease including left main coronary stenosis - Patient awaiting coronary artery bypass surgery - Non-cardiopulmonary limitation to exercise - Severe exercise intolerance - Neurological diseases. |
| ECG: echocardiography. MMSE: Mini-Mental State Evaluation. LVEF: Left ventricular ejection fraction. | | |

**Table S2:** Univariate correlation coefficient between CPET, cardiac hemodynamic and composite Z scores for cognitive functions.

| **n=35** | **Processing speed** | **Executive functioning** | **Verbal memory** |
| --- | --- | --- | --- |
| $\dot{V}$O_2peak_ (mL/min/kg) | -0.64*** | -0.74*** | 0.56*** |
| VT_1_ (mL/min/kg) | -0.54** | -0.69*** | 0.59*** |
| PPO (Watts) | -0.51** | -0.67*** | 0.54*** |
| O_2_ pulse  (mL O_2_/bpm) | -0.41* | -0.53** | 0.46** |
| HR_peak_ (bpm) | -0.52** | -0.72*** | 0.57*** |
| CO_peak_ (L/min) | -0.45** | -0.66*** | 0.35* |
| CI_peak_ (L/min/m^2^) | -0.56*** | -0.74*** | 0.42* |
| LCWi_peak_ (kg.m/m^2^) | -0.53** | -0.69*** | 0.37* |
| ** p<0.05, **p<0.01, ***p<0.001* | |  |  |

$\dot{V}$O_2peak_: cardiorespiratory fitness; VT_1_: V̇O_2_ at first ventilatory threshold; PPO: peak power output; HR: heart rate; CO: cardiac output; CI: cardiac index; LCWi: left cardiac work index.
