# Supplementary figures and images for: Cardiorespiratory Fitness Mediates Cognitive Performance in Chronic Heart Failure Patients and Heart Transplant Recipients
Source: Int J Environ Res Public Health. 2020 Nov 19;17(22):8591. doi: 10.3390/ijerph17228591 (PMC7699401; doi:10.3390/ijerph17228591)

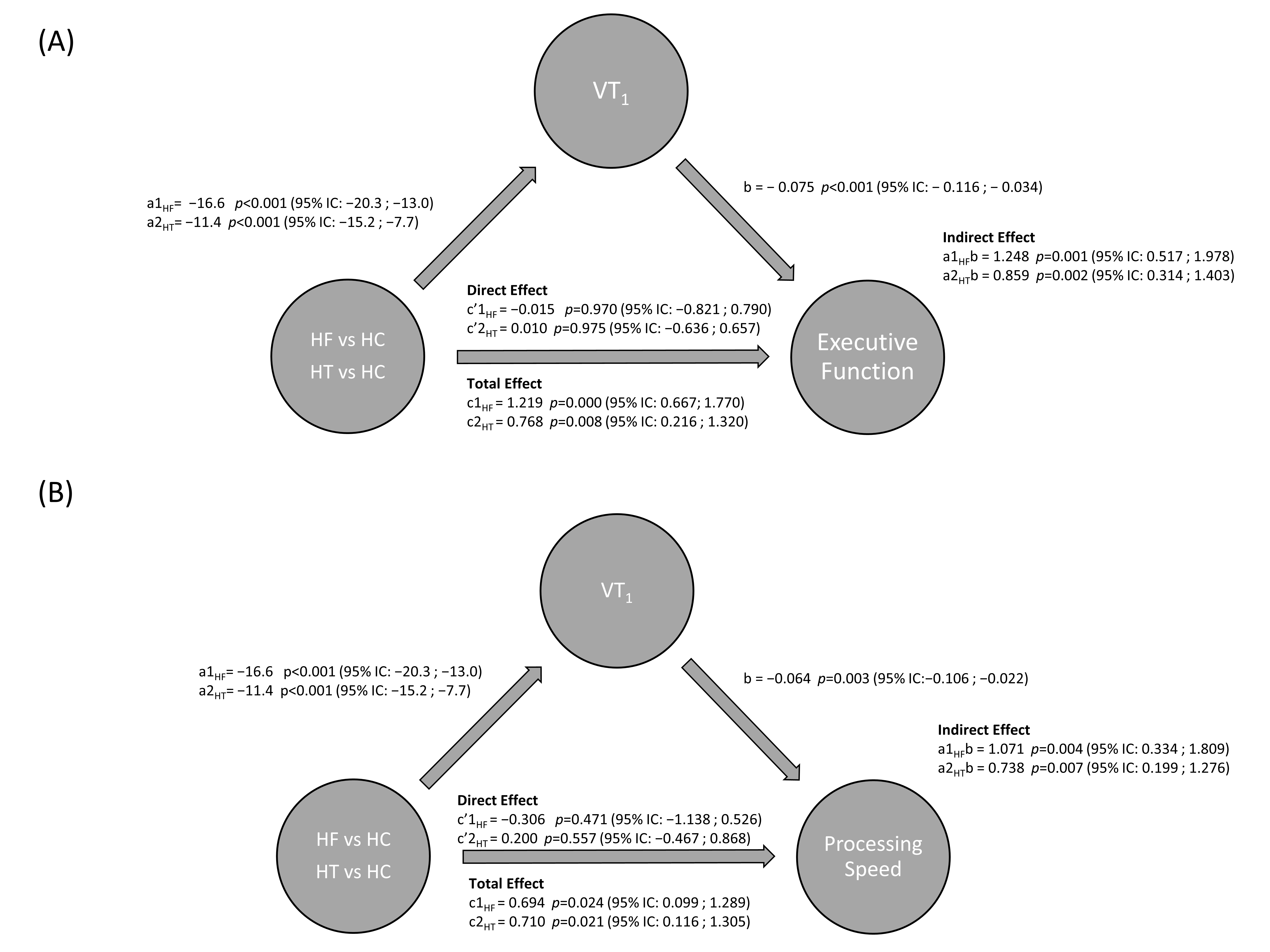

Supplement: Supplementary file 1 [file ijerph-17-08591-s001.zip › Fig S1.TIF]

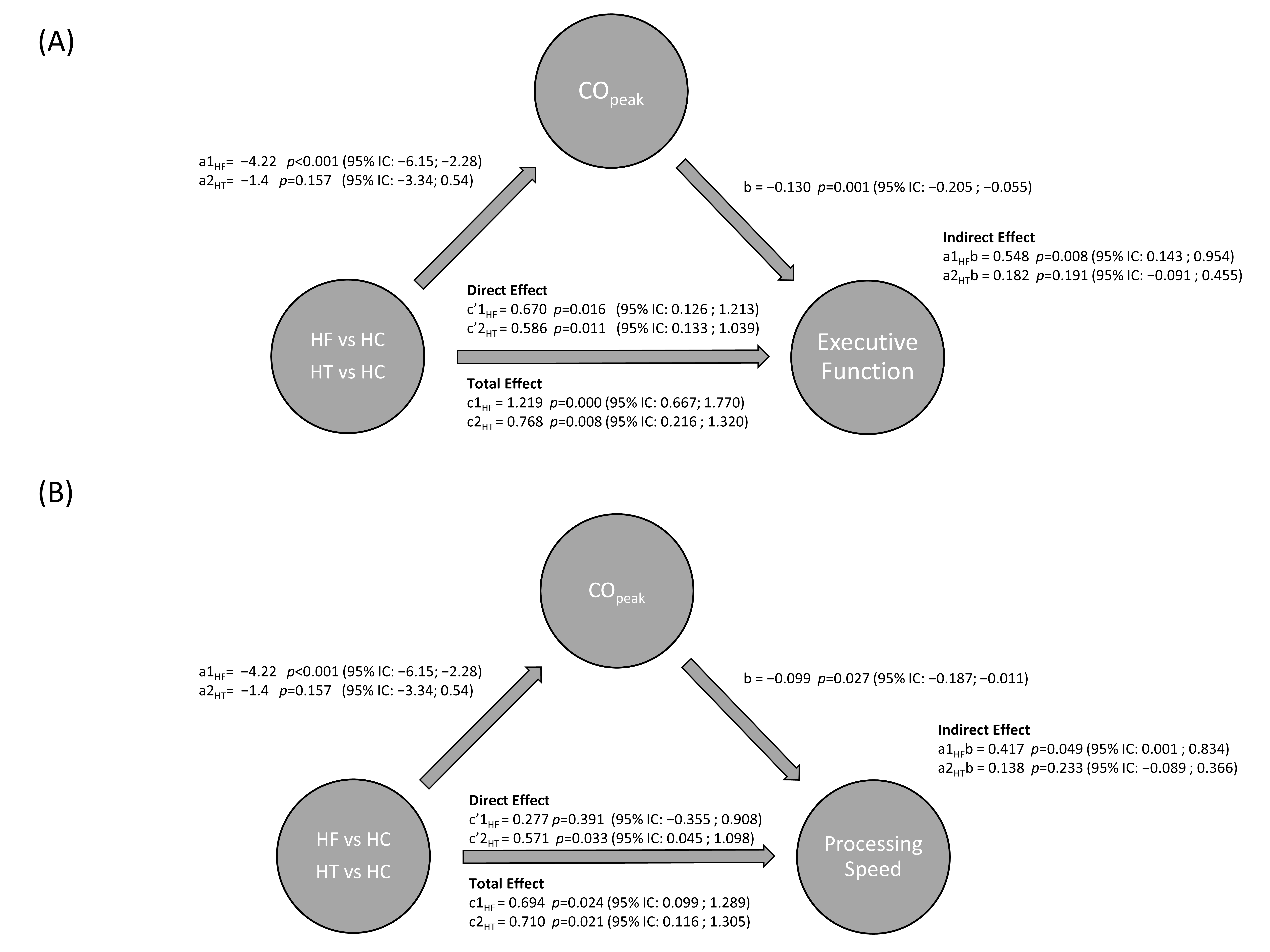

Supplement: Supplementary file 1 [file ijerph-17-08591-s001.zip › Fig S2.TIF]
